# CYRI proteins: controllers of actin dynamics in the cellular ‘eat vs walk’ decision

**DOI:** 10.1042/BST20221354

**Published:** 2023-03-09

**Authors:** Laura M. Machesky

**Affiliations:** 1 Department of Biochemistry, University of Cambridge, Tennis Court Road, Cambridge, U.K.; 2 CRUK Beatson Institute and Institute of Cancer Sciences, University of Glasgow, Glasgow, U.K.

**Keywords:** actin dynamics, cell migration, endocytosis, macropinocytosis, trafficking

## Abstract

Cells use actin-based protrusions not only to migrate, but also to sample their environment and take up liquids and particles, including nutrients, antigens and pathogens. Lamellipodia are sheet-like actin-based protrusions involved in sensing the substratum and directing cell migration. Related structures, macropinocytic cups, arise from lamellipodia ruffles and can take in large gulps of the surrounding medium. How cells regulate the balance between using lamellipodia for migration and macropinocytosis is not yet well understood. We recently identified CYRI proteins as RAC1-binding regulators of the dynamics of lamellipodia and macropinocytic events. This review discusses recent advances in our understanding of how cells regulate the balance between eating and walking by repurposing their actin cytoskeletons in response to environmental cues.

## Introduction

Actin-based protrusions at the plasma membrane allow the cell to explore space in all directions and to make new contacts with or even engulf samples of extracellular substances. By extending itself, the cell can actively pull and push against extracellular barriers to allow movement or it can take in nutrients to fuel its activity. The main machinery enabling actin-based protrusion at the plasma membrane is the 5-subunit Scar/WAVE complex (also termed WRC for WAVE regulatory complex). This complex interfaces with the 7-subunit Arp2/3 complex to generate branched actin filament structures that are mechanoresponsive and capable of force generation. Arp2/3 is activated by WRC in the presence of the active small GTPase RAC1 and by interaction with actin filaments to create a burst of dendritic actin filament branches that push against the plasma membrane, resulting in sheet-like extensions, termed lamellipodia. RAC1, WRC and Arp2/3 form a feedback loop to sustain dynamic actin reorganisation, which is orchestrated by many other signalling and actin-interacting proteins. We recently described negative regulators of this feedback loop, CYRI proteins, which also interact with RAC1 and display plasma membrane localisation through a myristoyl lipid anchor on their N-terminus [1]. By opposing WRC activation, CYRI proteins increase the dynamics of lamellipodia and provide cells with another level of control of their actin-based protrusions.

Related to lamellipodia are membrane ruffles, which occur when a lamellipodium lifts off the substratum. Ruffles can morph into cup-shaped structures that take up large gulps of extracellular fluid in a process called macropinocytosis. Macropinocytosis is mediated by large (0.5–10 μm) membrane cups that collapse into vesicles and bud into the cytoplasm from the plasma membrane. Macropinocytic structures are considerably larger than cups or pits formed in other types of endocytosis, including clathrin and caveolin-mediated endocytosis, which take place on the sub-micrometer scale (reviewed in [2]). Macropinocytic events frequently occur within the context of actin-driven membrane ruffles and high levels of activation of the RAC1 [3] and/or CDC42 [4] GTPase. Stages of macropinocytosis include the formation of an initial actin-driven protrusion, shaping into a cup and subsequent cup closure and engulfment. The order of recruitment and activation of various signalling and cytoskeletal molecules such as PtdIns3-kinase, RAC1, actin assembly and subsequent RAB5 recruitment helps to define these stages (for recent reviews see [5–8]).

Multiple types of cells perform macropinocytosis, including amoebas, cancer cells, immune cells, endothelial cells and mesenchymal cells. Endothelial cells can also perform stimulated macropinocytosis during angiogenesis, as vascular endothelial growth factor (VEGF) stimulates macropinocytosis of its own receptor, the VEGFR [4]. Epithelial cells are thought to perform relatively little or no macropinocytosis unless stimulated by growth factors such as epidermal growth factor (EGF), whereby they initiate ‘regulated macropinocytosis’. This may define an important difference between normal and cancer cells [8], which frequently perform ‘constitutive macropinocytosis’, even in the absence of stimulation. Constitutive macropinocytosis is thought to be driven by activating mutations in KRAS in pancreatic and other cancers [9]. It is likely that activating mutations in general of RAS, RAC and PI3-kinase signalling pathways could lead to constitutive macropinocytosis in multiple tumour types.

## Mechanisms of actin polymerisation driving macropinocytosis

**Figure 1.**
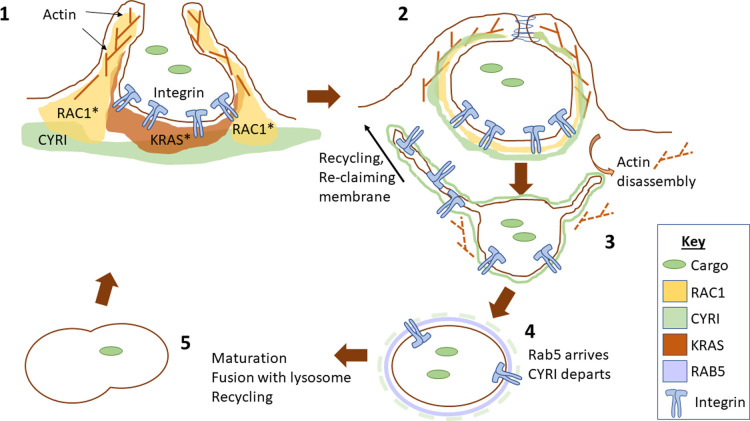
Illustration of the stages of macropinocytosis as described in the text.

It can be helpful to think of macropinocytosis as a series of stages, to place molecular events into the order in which they occur and to determine the function of the various cellular machinery employed (see Figure 1).

### Stage 1

Protrusion — Initiation of macropinocytosis begins with the protrusion of a membrane ruffle driven by WRC and Arp2/3-mediated actin assembly [10–15]. Protrusions can take the form of laterally or dorsally extending ruffles and in macrophages, they can be underpinned by actin tent-pole-like structures that aid closure [6,16]. Some models depict the actin nucleation-promoting protein N-WASP as a mediator of spiky protrusions in macropinocytic cups [5].

### Stage 2

Closure of the cup — Cup closure involves the fusion of the extended membrane into a vesicle or series of vesicles that are then taken into the cell. This requires remodelling and disassembly of the actin structures and is accompanied a transient increase in CYRI-A followed by a decrease in RAC1 signal at the membrane of the nascent macropinosome [14,17]. Both losses of CYRI and hyperactivation of RAC1 do not prevent the initial extension of ruffles, but prevent subsequent resolution or engulfment of macropinocytic cups, showing the need for dynamic actin remodelling. We hypothesise that a feedback loop of active RAC1 enhances the recruitment of the inhibitor, CYRI, which in turn mediates the dampening of the activation signal and allows closure. How or whether CYRI co-ordinates with RAC GTP exchange factor (GEF) and GTPase activating protein (GAP) proteins is not yet known. VAV1 could be interesting to explore in this context, as it has previously been implicated in macropinocytosis of bone morphogenetic protein receptors during *Drosophila* neural development [18,19].

### Stage 3

Engulfment of the macropinosome — Stenmark and colleagues describe two main stages for macropinosome engulfment into mammalian cells, with the first being a closed vesicle that is highly deformed by the cortical actin network. The vesicle eventually squeezes through the actin network and becomes rounded as it traverses deeper into the cytoplasm [20,21]. The protein Phafin2 regulates the maturation of macropinosomes by detecting the presence of PtdIns3P and PtdIns4P simultaneously and promoting the reorganisation of the actin cytoskeleton to free the macropinosome from its actin network [21]. During early engulfment, macropinosomes undergo explosive tubulation, presumably to retrieve membrane and receptors back to the plasma membrane [22].

### Stage 4

Movement into the cytoplasm — After capture by microtubules that penetrate through the cell cortex, macropinosomes traverse through the dense actin network and deeper into the cell in a dynein- and microtubule-dependent manner [20]. JIP3 and Arf6 also regulate this phase of the movement, by scaffolding the interaction. Myosin-II regulates macropinosome deformation as it squeezes through the dense actin network and may be required for effective passage [20].

### Stage 5

Fusion with lysosomes — Finally, the macropinosomes fuse with other endocytic organelles and eventually with lysosomes in a process mediated by phosphoinositide lipids and their contents are broken down into basic chemical building blocks to allow the cell to use the energy from proteins, lipids and sugars thus engulfed (reviewed in [23]).

## Motility-related functions of macropinocytosis

Perhaps the most well-understood function of macropinocytosis is nutrient uptake. Macropinocytosis allows cells to use alternative and larger-sized nutrients including lipids, proteins and cell fragments. This function is well known to fuel cancer growth (reviewed in [8]), as tumours frequently become a starvation environment that is poorly vascularised and therefore short of glucose and amino acids. In particular, pancreatic cancer is almost always driven by mutant-activated KRas, which leads to the hyperactivation of signalling pathways that trigger macropinocytosis, promoting tumour aggressiveness and survival [9].

Macropinocytosis is also connected with cell migration, both directly via actin dynamics and indirectly via receptor trafficking. The best-understood example is in *Dictyostelium*, where PtdIns3-kinase drives the formation of macropinocytic cups that generally oppose migration and fuel the axenic growth of cells [12,13]. *Dictyostelium* normally feed on bacteria and as such prefer solid growth substrata. However, common laboratory strains readily grow axenically in liquid culture. Recently, the secret to survival in liquid medium was described as an activating mutation in the RAS GAP, NF1, in common laboratory strains [24]. *Dictyostelium* RAS strongly localises to the centre of macropinocytic cups in these axenic strains, in a zone that is PtdIns3-rich. NF1 is proposed to restrict the size and frequency of initiation of such zones by controlling RAS activity [24] and mutation or loss can lead to exaggerated macropinocytosis.

*Dictyostelium* amoebas use separate structures for macropinocytosis and migration and that these can work in opposition [12,25]. It is unclear how macropinocytosis opposes migration in *Dictyostelium*, but it might be due to the repurposing of branched actin filaments away from lamellipodia and towards macropinosomes. Arp2/3 complex may simply become limiting for making new branched actin, or even actin itself may be limiting, as is found in budding yeast, where actin cables and actin patches play an opposing role limited by the availability of actin and actin-binding proteins [26]. Another possibility is that RAC1 or other signalling molecules are similarly repurposed to macropinosomes and away from migratory actin structures. In mammalian cells, it isn't clear whether macropinocytosis and migration play opposing roles, but deletion of the CYRI protein caused a reciprocal inhibition of macropinocytosis and enhancement of migration [1,14], suggesting that at least in some cell types this might hold true.

Evidence is building that macropinocytosis is an important controller of membrane trafficking for certain cell surface proteins and receptors. We recently found that disabling macropinocytosis by depletion of CYRI proteins led to enhanced surface integrin accumulation, enhancing invasiveness and adhesion of cancer cells [14]. Integrin is a known cargo of circular dorsal ruffles [27], but other cargo such as BMP receptors [18,19] and platelet-derived growth factor receptors also traffic via macropinocytosis [28]. Whether this role for CYRI and cargo specificity is shared among various cell types and cancers needs further investigation. How much of a contribution to receptor trafficking does macropinocytosis make, relative to other forms of endocytosis? This question remains unanswered as does the question of whether specific surface receptors are preferentially trafficked by macropinocytosis. Specific trafficking could be co-ordinated, for example, by membrane lipid composition or even actin assembly, e.g. as previously shown for WASH [29], but this remains to be discovered.

Another major function of macropinocytosis is the rapid regulation of membrane surface area. Dendritic cells faced with navigation of narrow spaces will rapidly macropinocytose membrane to allow squeezing through tight places [30]. The trigger for this rapid adjustment is thought to be barotaxis, or the sensing of pressure in the environment. Another situation where the membrane is rapidly internalised is to facilitate neuron retraction [31]. Macropinocytosis has also been observed to aid retraction of the rear of *Xenopus* embryo endoderm cells, which has been termed ingression-type migration [32]. Multiple lines of evidence suggest that macropinocytosis represents an important mechanism for cells to engulf a large proportion of their membrane quickly, in order to change their size or shape rapidly.

## The role of CYRI proteins in macropinocytosis vs motility

**Figure 2.**
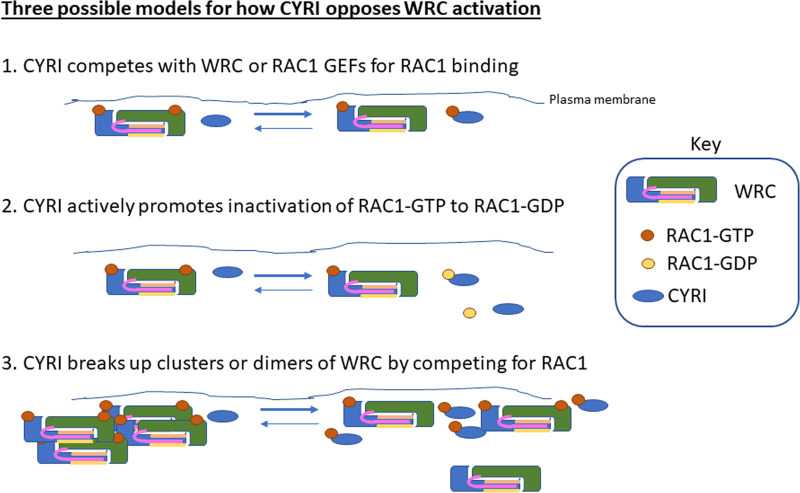
Three possible models for how CYRI could oppose RAC1-mediated activation of WRC. In Model 1, CYRI competes with WRC and/or RAC GTP exchange factor (GEF) proteins to limit the amount of active RAC1 available for the activation of WRC. In Model 2, CYRI acts as a GTPase activating protein (GAP) to actively promote the conversion of active GTP-RAC1 to inactive GDP-RAC1. In Model 3, CYRI prevents or destabilises active clusters or dimers of WRC by limiting the amount of available RAC1-GTP. None of these models has yet been proven or disproven.

CYRI proteins are evolutionarily conserved proteins that directly interact with RAC1 and oppose RAC1-mediated activation of WRC [1]. CYRI proteins bind directly to RAC1, via a similar site as the CYFIP subunit of the WRC [1,33] (Figure 2). This interaction promotes a decrease in RAC1 activity in cells overexpressing CYRI, by an unknown mechanism [1]. Small GTPases like RAC1 are normally regulated by GEFs, which enhance guanine nucleotide exchange and promote activation via GTP association. Conversely, small GTPases are inactivated by GAPs, which promote the hydrolysis of GTP to GDP. There is currently no evidence that CYRI acts as a GAP for RAC1, but this possibility warrants further investigation. Possible mechanisms for how CYRI opposes RAC1-mediated WRC activation include: sequestering RAC1 away from activation by GEFs or from interaction with WRC (Figure 2, Point 1); Furthermore, WRC requires two active RAC1 molecules simultaneously [34], so if CYRI can deactivate or remove one of these, it could be a potent and dynamic regulator of WRC (Figure 2, Point 2); WRC is thought to require dimerisation at the plasma membrane, or even to form higher order clusters [35], so CYRI might function to break up the clusters and thus make active WRC more dynamic by reducing the lifetime of activation (Figure 2, Point 3).

We recently discovered that CYRI proteins are key controllers of macropinocytosis [14]. The signals that recruit CYRI to nascent macropinocytic cups and then regulate its departure as the cups close are unknown, but possibilities include RAC1 activation, phosphorylation, lipid modification [1] and association with protein or lipid components at the plasma membrane. Mammalian cells have two isoforms of CYRI, CYRI-A and CYRI-B. CYRI-B is fairly universally expressed, while CYRI-A is much more restricted and is enriched in some blood cell lineages, Langerhans cells and sarcoma cells (Human Protein Atlas). Almost nothing is known about whether CYRI-A and CYRI-B have unique functions, but the knockdown of each of them seems to have additive functions in sarcoma cells [14]. CYRI-A and CYRI-B can form homo and heterodimers, which oppose RAC1 binding [36], suggesting that regulated dimer formation might control localisation. No direct binding partners besides RAC1 have been described for CYRI so far, so it remains open whether protein or lipid partners could regulate its association with actin and membranes. CYRI-A and CYRI-B may be differently regulated, as fluorescent fusion constructs of each show differential recruitment to the plasma membrane, with CYRI-B being constitutively associated and CYRI-A being much more transiently recruited [14]. However, because the constructs used to study CYRI dynamics are fusion proteins with an internally inserted fluorescent protein [14], further studies are needed to confirm the dynamics.

Could CYRI proteins be coincidence detectors of RAC1 and a lipid at the plasma membrane surface? This could explain the differential recruitment of CYRI-A and CYRI-B. The proteins and lipids that define macropinocytic cups and lamellipodia are largely overlapping. However, in *Dictyostelium*, the presence of PtdIns 3,4,5 P3 may distinguish macropinocytic cups from lamellipodia protrusions [7,12,13,37]. This discovery was key to understanding that PtdIns3-kinase, while involved in migration, is much more crucial for macropinocytosis.

CYRI proteins participate in enhancing lamellipodia dynamics and macropinocytosis, but do they also regulate other forms of plasma membrane-mediated cellular engulfment — e.g. microendocytosis or phagocytosis? Phagocytosis is a likely candidate, as CYRI seems to offer cells protection against the uptake of certain pathogens such as *Salmonella*, which enter using actin-based protrusions [33]. Evidence does not support a role in small nanoscale endocytosis, either clathrin or non-clathrin [14], but more in-depth studies are needed to be conclusive. It will be interesting to test the role of CYRI in the entry of various pathogens, to know whether all RAC1-dependent entry mechanisms also involve CYRI proteins or only a subset.

CYRI proteins may have evolved to protect cells against the low-level engagement of RAC1-mediated ruffling, such as induced by contact with some pathogens or foreign particles. By having a buffer for RAC1-mediated protrusions, CYRI makes cell membranes more dynamic and more resistant to stimulation. This perhaps suggests a complex role for CYRI in the immune system, as it could be protective for pathogen entry, but if it has a positive role in the resolution of macropinocytosis, it could also promote pathogen entry via macropinocytosis.

In summary, CYRI proteins increase cell membrane dynamics and thus shorten the lifespan of protrusions, while allowing cells to make crucial decisions about whether to use their actin cytoskeleton to migrate or to take samples of the environment. CYRI proteins sit at the centre of the eat vs walk decision and future research will hopefully reveal new mechanisms and connections between CYRI proteins and the cellular machinery controlling these processes. CYRI is clearly only one small player in a complex web of feedback loops controlling the cytoskeletal and membrane changes taking place during lamellipodia and macropinocytic cup dynamics. However, the study of CYRI holds promise to reveal new aspects of these processes.

## Perspectives

- Gaining a better understanding of how cells control the balance between the assembly of lamellipodia for migration or for macropinocytosis is fundamentally important for understanding how cells balance energy-consuming processes, such as migration, with energy uptake and production. It also can potentially provide insights into how to control these processes or exploit them for therapeutic purposes. Delivery of nanoparticle drugs via macropinocytosis might provide enhanced targeting of chemotherapeutics to cancer cells (reviewed in [38–40]) and there is some interest in the process of death by macropinocytosis termed methuosis [41,42] as a way of controlling cell death. However, control of macropinocytosis and migration is complex, and many unanswered questions remain.
- Even though macropinocytosis was first described in the 1930s (reviewed in [7]), the field is still actively discovering new players and mechanisms. Understanding how macropinocytosis and migration are co-ordinated by cells promises to offer new insights into areas such as how cells control energy supply and demand in challenging environments.
- Future research should aim to identify the common basic principles and control mechanisms that are conserved and fundamental in macropinocytosis. Additionally, if we better understand the specialisations related to different cancer mutations, cell types, signalling or environmental cues, we have a chance to uncover unique opportunities to understand or exploit macropinocytosis and the eat vs walk machinery to combat cancer, pathogens or other diseases.

## Open Access

Open access for this article was enabled by the participation of the University of Cambridge in an all-inclusive *Read & Publish* agreement with Portland Press and the Biochemical Society under a transformative agreement with JISC.

## Abbreviations

GAPs: GTPase activating proteins
GEFs: GTP exchange factors
VEGF: vascular endothelial growth factor
